# Futuristic Novel Therapeutic Approaches in the Treatment of Rheumatoid Arthritis

**DOI:** 10.7759/cureus.49738

**Published:** 2023-11-30

**Authors:** Vikramaditya Rai, Nirmal Patel, Simi R Mammen, Sachin M Chaudhary, Sanan Arshad, Shahzad W Munazzam

**Affiliations:** 1 Orthopedics, Dr. Rajendra Prasad Government Medical College, Kangra, IND; 2 Internal Medicine, St. George’s University School of Medicine, Saint George's, GRD; 3 Internal Medicine, Medical University of Sofia, Sofia, BGR; 4 Internal Medicine, Gujarat Cancer Society (GCS) Medical College, Hospital and Research Centre, Ahmedabad, IND; 5 Internal Medicine, Hayatabad Medical Complex, Peshawar, PAK; 6 Orthopedic Surgery, Hayatabad Medical Complex, Peshawar, PAK

**Keywords:** small molecule inhibitors, cell-based therapies, autoimmune arthritis, liposomal immunotherapy, interneuron crosstalk, targeted biologics, emerging therapeutics, rheumatoid arthritis

## Abstract

Rheumatoid arthritis (RA) is a chronic autoimmune disease characterized by systemic inflammation and joint destruction, leading to significant morbidity and reduced quality of life. Although significant progress has been made in the management of RA over the past few decades, many patients still fail to respond adequately to currently available therapies. This article aims to review the current landscape of RA treatment and explore potential novel therapeutic approaches that hold promise for the future. Advances in our understanding of the underlying pathogenesis of the disease have led to the identification of new targets and the development of innovative treatment strategies. This review focuses on emerging therapies including small molecule inhibitors, targeted biologics, cell-based therapies, and gene editing technologies that have shown potential in preclinical and early clinical trials. Additionally, we discuss the challenges and opportunities associated with the use of these new approaches in the treatment of RA. By elucidating the future of novel therapeutic approaches, this article provides insights that can guide clinicians and researchers in their efforts to improve outcomes for patients with RA.

## Introduction and background

Rheumatoid arthritis (RA) is an intricate autoimmune condition characterized by persistent inflammation in the synovial joints, resulting in gradual deterioration of the small joints, impairment of bodily functions, and considerable morbidity. Its prevalence is estimated to be around 0.5-1% of the global population and is more commonly observed in females than males [1]. Approximately 1.3 million individuals in the United States exclusively encounter RA, signifying it holds notable importance in the realm of health [2]. Nevertheless, innovations in medical investigation have given rise to the emergence of numerous therapeutic alternatives that can effectively control the indications and decelerate the advancement of the condition. The current approach to managing RA primarily revolves around the administration of disease-modifying anti-rheumatic drugs (DMARDs) such as methotrexate, biologic therapies that encompass tumor necrosis factor (TNF) inhibitors, and the utilization of glucocorticoids [3]. These drugs decrease inflammation and pain, reduce tissue damage, and slow the progression of the disease. The treatment plan includes a checkup schedule to review progress and watch for side effects. However, a notable proportion of individuals still fail to attain remission or experience adverse effects that are intolerable.

The initial approach to managing RA typically involves the administration of DMARDs. These pharmaceutical agents function by suppressing the immune system to mitigate inflammation and avert damage to the joints. Methotrexate is the most commonly recommended DMARD for RA. This medication has exhibited efficacy in diminishing joint inflammation and preventing structural harm. It can be administered orally or via injections and has demonstrated substantial effectiveness in managing symptoms and enhancing the long-term prognosis for patients. Nevertheless, a considerable proportion of patients do not respond to methotrexate or experience unfavorable reactions, necessitating alternative treatment options [4]. In addition to methotrexate, other DMARDs like sulfasalazine, leflunomide, and hydroxychloroquine are also employed as therapeutic alternatives. These medications are frequently prescribed in combination to achieve optimal effectiveness. However, they may induce side effects and necessitate regular monitoring to identify any adverse reactions.

In instances where the effectiveness of DMARDs alone is lacking in the management of symptoms, it may be advisable to consider the utilization of biologic response modifiers, also known as biologics. These biologic therapies have brought about a significant transformation in the treatment of RA by specifically targeting the molecules that are implicated in the inflammatory cascade [5]. Noteworthy drugs such as TNF inhibitors, interleukin-6 (IL-6) inhibitors, and Janus kinase (JAK) inhibitors have demonstrated promising outcomes [1]. The mechanism of action of TNF inhibitors, like adalimumab, etanercept, and infliximab, involves the suppression of TNF activity, a protein that plays a crucial role in the inflammatory process. IL-6 inhibitors such as tocilizumab and sarilumab target IL-6, another protein that is involved in the inflammatory response. JAK inhibitors like tofacitinib and baricitinib exert their effects by obstructing the activity of enzymes known as JAKs, which are implicated in the signaling pathways that contribute to inflammation in RA [6].

Biologics, despite demonstrating significant effectiveness, generally carry a higher price tag compared to traditional DMARDs. Furthermore, they may lead to adverse events such as heightened susceptibility to infections, hepatotoxicity, and cutaneous reactions. Consequently, meticulous surveillance and routine hematological examinations are imperative during the administration of these medications.

In recent times, there has been an escalating curiosity regarding the utilization of holistic techniques and supplementary therapies in the management of symptoms associated with RA [7]. These approaches encompass exercise regimens, physical rehabilitation, occupational therapy, and counseling, all of which aid patients in adapting to the psychological dimensions of enduring chronic pain. Additionally, altering one's dietary habits and incorporating supplements like fish oil and turmeric have exhibited certain advantages in terms of mitigating inflammation and enhancing overall well-being [8].

## Review

Emerging therapeutic approaches

Advancements in therapeutic approaches and the comprehension of pathogenesis have resulted in an enhanced forecast and a probable state of remission for RA. However, a certain subgroup of patients has exhibited only partial or non-response to traditional and biologic treatments, thereby emphasizing the necessity for novel therapeutic interventions [9]. The occurrence of specific interactions between genotypes, environmental risk factors, and autoimmunity implies a molecular foundation for the breach of tolerance and the initiation of pathogenic immune reactions in extra-articular mucosal sites early on in the course of disease development [1]. Furthermore, the dysregulation of gene expression at the epigenetic level also leads to modified immune responses and the development of the disease [10,11]. Based on these hypotheses, numerous clinical studies have been initiated by researchers worldwide (Table 1).

**Table 1:** List of some of the ongoing clinical trials on various treatment therapies for RA (https://clinicaltrials.gov). RA, rheumatoid arthritis; DMARDs, disease-modifying antirheumatic drugs; TNF, tumor necrotic factor; JAK, Janus kinase; MPCs, mesenchymal precursor cells; RAMBA, RA memory B cells and Abatacept; anti-CCP, anti-cyclic citrullinated peptide; HAQ, health assessment questionnaire; VTEs, venous thromboembolic events; DRL-R, diagnostic reference level-rituximab; FU, Florida University; EU, European Union

S. No.	Title	Clinical Trial No.	Study Phase	Purpose
1.	Strategy to prevent the onset of clinically apparent RA.	NCT02603146 (https://clinicaltrials.gov)	2	To determine if hydroxychloroquine is safe and effective for the prevention of future onset of RA in individuals who have an elevation of an auto-antibody, anti-CCP.
2.	A study of RoActemra/Actemra (Tocilizumab) in comparison to Etanercept in patients with RA and cardiovascular disease risk factors.	NCT01331837 (https://clinicaltrials.gov)	4	This multicenter study will evaluate the rate of cardiovascular events of the drug in comparison to Etanercept in patients with RA.
3.	A single IV infusion of allogenic MPCs in patients with RA and incomplete response to atleast one TNF alpha inhibitor.	NCT01851070 (https://clinicaltrials.gov)	2	A multicenter study to evaluate the safety, tolerability, and feasibility of a single intravenous injection of allogeneic MPCs compared to placebo at 12 weeks post-infusion in the treatment of patients with active RA who have received methotrexate +/- other DMARDs for atleast six months prior to screening and who have had an incomplete response to atleast one TNF-alpha inhibitor.
4.	A study comparing the safety of Tofacitinib versus TNF inhibitor in patients with RA.	NCT02092467 (https://clinicaltrials.gov)	4	To compare the safety of tofacitinib to JAK inhibitor versus TNF inhibitor with respect to major cardiovascular adverse events and malignancies, excluding non-melanoma skin cancers, when given to subjects with RA.
5.	RA-pro pragmatic trial.	NCT04692493 (https://clinicaltrials.gov)	2	To examine if the strategy starting with the addition of a tsDMARD, oral targeted molecule medications will lead to greater improvement in HAQ compared with the alternate approach of the addition of a non-TNF-biologic.
6.	RAMBA.	NCT03652961 (https://clinicaltrials.gov)	4	To assess changes in the immune profile in response to treatment with intravenous Abatacept adults with RA who are naive to biologic DMARDs.
7.	The effects of Acthar on synovial inflammation in RA.	NCT03511625 (https://clinicaltrials.gov)	3	Patients will receive either Depo Medrol or Acthar treatment for synovial inflammation. This will be done by synovial biopsy, blood draws, synovial fluid aspiration, and physician assessments before/after initiating treatment.
8.	A study to compare the response to treatment with Abatacept versus Adalimumab, on background methotrexate, in adults with early, seropositive, and shared Epitope-positive RA and an inadequate response to methotrexate.	NCT04909801 (https://clinicaltrials.gov)	3	To evaluate the superiority in the efficacy of Abatacept compared with Adalimumab on background methotrexate in adults with RA having inadequate response to methotrexate.
9.	A study of Barcitinib (LY3009104) in participants with RA (RA-BRIDGE).	NCT03915964 (https://clinicaltrials.gov)	4	To compare the safety of Barcitinib versus TNF inhibitors with respect to VTEs when given to participants with RA.
10.	Long-term extension study to assess the safety and efficacy of filgotinib in adults with RA (FINCH4).	NCT03025308 (https://clinicaltrials.gov)	3	To evaluate the long-term safety and tolerability of Figotinib in participants who have completed one of the parent studies of Figotinib in RA.
11.	A study of Peresolimab (LY3462817) in participants with moderately-to-severely active RA (RESOLUTION-1).	NCT05516758 (https://clinicaltrials.gov)	3	To study the safety and efficacy of Peresolimab in adult participants with moderate-to-severe RA.
12.	A phase III transition study of DRL-R to reference medical products (RI-01-007).	NCT04268771 (https://clinicaltrials.gov)	3	To assess the immunogenicity and safety of transitioning subjects with RA to DRL-RI from US-rituximab/FU-Rituximab to continued treatment with US-rituximab/EU-rituximab

Small Molecule Inhibitors

Over the course of the previous decade, various inhibitors composed of small molecules have been developed to target specific intracellular pathways known to play a role in the pathogenesis of RA. Among these inhibitors, JAK inhibitors, such as tofacitinib and baricitinib, have exhibited effectiveness in reducing the activity of the disease and enhancing the outcomes reported by patients. However, recent concerns about the use of JAK inhibitors involved embolism and thrombosis in addition to skin neoplasms. Future advancements in JAK inhibitors are currently centered around augmenting their specificity and effectiveness, while simultaneously minimizing any adverse effects that may arise (Table 1; NCT02092467; NCT03915964).

A recent study explored the application of the small molecule inhibitor XST (xanthohumol solubilized in Tween-80) in the treatment of RA [12]. The researchers discovered that XST notably diminished the swelling and inflammation of joints in a mouse model of RA. Furthermore, this inhibitor hindered the activity of enzymes involved in the production of pro-inflammatory molecules, thereby suppressing the immune response and preventing any harm to the joints.

Another investigation focused on the small molecule inhibitor known as tasquinimod. The scientists discovered that tasquinimod mitigated joint inflammation and damage in a mouse model of RA. The inhibitor functioned by impeding the activity of specific immune cells and diminishing the production of pro-inflammatory cytokines [13].

Both investigations emphasize the potential of small molecule inhibitors in the management of RA. These inhibitors offer benefits such as enhanced oral bioavailability, reduced cost, and improved patient adherence when compared to biological agents. Nevertheless, further examination is necessary to assess their effectiveness and safety in clinical trials involving human subjects.

Targeted Biologics

The successful utilization of TNF inhibitors has paved the path for the advancement of innovative biologic therapies that aim at other crucial cytokines and immune cells that are involved in the pathogenesis of RA (inhibitors of IL-6, such as tocilizumab and sarilumab) and have demonstrated their effectiveness as both standalone treatments and when used in conjunction with methotrexate. Additionally, inhibitors that target IL-17A, IL-23, B cells, and granulocyte-macrophage colony-stimulating factor (GM-CSF) have exhibited promising outcomes in early clinical trials. Several ongoing clinical trials are investigating the efficacy and tolerability of targeted biologics such as abatacept, acthar, peresolimab, and rituximab (Table 1; NCT03652961; NCT03511625; NCT05516758; NCT04268771). It is imperative to point here that all biologics suppress the immune system and increase the risk of infections such as upper respiratory infections, pneumonia, urinary tract infections, skin infections, and other opportunistic infections.

Cell-Based Therapies

The notion of employing cell-based therapies in the context of RA entails the modification or manipulation of immune cells in order to reinstate immune homeostasis. Early-phase clinical trials have yielded encouraging outcomes with regard to the utilization of mesenchymal stem cell (MSC) therapy, as it effectively modulates the inflammatory response and facilitates tissue repair (Table 1; NCT01851070). Another strategy involves chimeric antigen receptor T-cell (CAR-T) therapy, which focuses on autoreactive immune cells. Researchers are currently in the process of developing a universal CAR-T therapy that can identify and target autoreactive lymphocytes using major epitope peptides, although additional investigations are still necessary. Furthermore, the adoptive transfer of CAR-anti-inflammatory regulatory T-cells (Tregs) has demonstrated promise in terms of effectively reducing inflammation and treating autoimmunity. Through this thorough examination, the authors aim to provide an all-encompassing comprehension of the current state of research on this subject, identify areas that necessitate further study, and promote the advancement of CAR-T therapy as a viable option for the treatment of RA [14]. 

Natural killer (NK) cells are a type of immune cell that can target and kill abnormal cells, including cancer cells and infected cells. In RA, NK cells have been found to have impaired function. A clinical study found that infusion of ex vivo-expanded NK cells in patients with RA was safe and reduced disease activity [15].

Overall, while cell-based therapies offer promising results for RA, more large-scale clinical trials are needed to validate their efficacy and safety.

Gene Editing Technologies

However, there are several challenges that must be addressed before gene editing can be implemented in a clinical environment, including the potential for off-target effects and the need for effective delivery mechanisms. A recent study focused on the application of gene editing technology to modify T-cells in patients with RA. The researchers employed a technique known as lentiviral-mediated CAR-T gene transduction to modify T-cells, with the aim of targeting and eliminating cells that contribute to inflammation in RA. These findings indicate that the utilization of gene editing in this manner could present a promising therapeutic strategy for the treatment of RA. The advancements in gene editing technologies have opened up exciting new possibilities in the realm of RA treatment. The Clustered Regularly Interspaced Short Palindromic Repeats (CRISPR)-Cas9 system, in particular, allows for precise modification of disease-associated genes, potentially leading to long-lasting therapeutic effects [16]. MYC, FOXO1 gene, SNP rs6927172, TNFAIP3, OLIG3 gene, and miR-155 have been suggested as appropriate candidates for CRISPR-Cas9 treatment. Conversely, there are numerous ethical and practical challenges to implementing gene editing in clinical practice in the current scenario.

Understanding the interneuron crosstalk: Recent research has unveiled that adenosine triphosphate (ATP) is secreted within the joints, a phenomenon that has the potential to exacerbate or even induce inflammatory responses. A hypothesis was put forth by scientists, postulating that neural crosstalk might be accountable for the manifestation of distal inflammation in mice [17]. In order to explore this notion, the sensory neural circuits connecting the left and right ankle joints were deliberately severed. Consequently, it was discovered that the transmission of the inflammatory signal from one joint to another occurs via a sensory neuron connection facilitated by the spinal cord. Notably, an escalation in ATP levels was observed in both joints, thus serving as a trigger for inflammation. Hence, the inhibition of this pathway holds great promise for mitigating the inflammatory response, thereby rendering it a noteworthy target for therapeutic intervention.

Poly(lactic-co-glycolic acid) all-trans retinoic acid microparticles: A recent report has presented an immunoregulatory approach that is centered around the sustained release of all-trans retinoic acid (ATRA) localized to the joints [18]. This approach serves to modulate immune activation at the local level and enhance disease-protective T-cells, ultimately leading to systemic disease control. It has been observed that ATRA induces a distinct chromatin landscape in T-cells, which is correlated with an increase in the differentiation of naïve T-cells into Treg, as well as the suppression of Treg destabilization. In arthritic mouse joints, poly-(lactic-co-glycolic) acid (PLGA)-based biodegradable microparticles encapsulating ATRA (PLGA-ATRA MP) exhibit sustained release and are retained following intra-articular (IA) injection. Moreover, IA PLGA-ATRA MP has been found to enhance the migration of Treg, leading to reduced inflammation and modification of disease in both injected and uninjected joints. This same effect can also be achieved through IA injection of Treg. In the SKG and collagen-induced arthritis mouse models of autoimmune arthritis, PLGA-ATRA MP has demonstrated the ability to decrease proteoglycan loss and bone erosions. Systemic disease modulation by PLGA-ATRA MP is observed to be strikingly devoid of any concurrent generalized immune suppression. The potential for PLGA-ATRA MP to be harnessed as a disease-modifying agent for autoimmune arthritis is evident.

Targeting tumor protein p63: Recently, a cohort of scientists has made a significant revelation concerning the interaction between methotrexate and tumor protein p63 (TP63) in CD4+ T-cells. In order to comprehend the impact of methotrexate on gene expression, the researchers employed the utilization of DNA microarray profiling on CD4+ T-cells obtained from patients suffering from RA [19]. Additionally, they implemented gene knockdown, a molecular technique to suppress a specific target gene, along with RNA sequencing (RNA-seq) to authenticate gene function. Through their investigations, the researchers discovered that TAp63, an isoform of TP63, exhibited a notable level of expression in both human and mouse Th17 cells. The data obtained from RNA-seq and gene knockdown procedures further unveiled that TAp63 successfully targeted another gene, FOXP3, which acts as the master regulator of Treg cells. A remarkable increase in the expression of FoxP3 protein was observed when TAp63 was "knocked down" in Treg cells. By conducting a reporter assay, the researchers were able to establish that TAp63 was binding to the FOXP3 enhancer and suppressed its functionality. Collectively, these intriguing findings suggest a complex association between TAp63 and the delicate equilibrium of Th17 and Treg cell differentiation. Thus, the inhibition of TAp63 may potentially augment the suppressive capabilities of Treg cells, thereby restricting the manifestation of autoimmune RA. The discovery of these outcomes elucidates a resilient mechanism through which methotrexate exerts its therapeutic effects, while also highlighting the preservation potential of Treg cells in the context of RA. Furthermore, these findings underscore the prospective value of TAp63 as a novel target for therapeutic intervention in RA.

Targeting NFATC1 gene: In this investigation, the scholars examined the potentiality of a therapeutic approach that focuses on the mechanisms associated with the differentiation procedure of osteoblasts, which dissolve bones through enzymatic reactions [20,21]. Initially, it was ascertained that a super enhancer (SE) is generated in close proximity to the NFATC1 gene, acknowledged as a pivotal element in osteoblast formation, and is exclusively produced in osteoblasts. Furthermore, it was validated that an enhancer RNA, classified as a form of non-encoded RNA, is generated within the NFATC1 SE during the process of osteoblastic cell formation. Non-encoded RNA does not encode proteins but assumes a significant role in the regulation of gene expression. Specifically, owing to the molecular sequence's specificity, it can be readily targeted for therapeutic intervention.

Indeed, it has been ascertained that the interference with NFATC1 SE RNA effectively hinders the development of osteoblasts. Consequently, it has been determined that targeting NFATC1 SE RNA, which emerges during osteoblast differentiation, can be employed as a viable treatment strategy.

This investigation serves as the pioneer in identifying SEs and SE-eRNAs within human osteoclasts, thereby enhancing our comprehension of human osteoclast biology. As a result, novel therapeutic approaches for pathological bone deterioration in humans can now be explored.

Liposomal immunotherapy

The regulation of autoimmune disease that is specific to antigens is a significant objective. In the case of seropositive RA, T-cells contribute to the maturation of autoreactive B cells, resulting in the development of a citrullinated (Cit) antigen-specific immune response [22]. This immune response produces RA-specific V domain glycosylated anti-Cit protein antibodies (ACPA VDG) prior to the onset of arthritis. The administration of antigens in low or escalating amounts under "sub-immunogenic" conditions promotes tolerance. In this study, the safety, pharmacokinetics, and immunological and clinical effects of s.c. DEN-181 were investigated. DEN-181 consists of liposomes that encapsulate self-peptide collagen II259-273 (CII) and the NF-κB inhibitor 1,25-dihydroxycholecalciferol.

A phase I trial was conducted to evaluate the effects of low, medium, and high doses of DEN-181 on autoreactive T-cell responses, cytokines, and ACPA in RA patients on methotrexate [23]. The trial employed a double-blind, placebo-controlled design and utilized a single-ascending-dose approach. The trial included 17 ACPA-positive RA patients who were HLA-DRB1*04:01+ or *01:01+. The results showed that DEN-181 was well tolerated. In patients who received medium and high doses of DEN-181, there was a decrease in Cit-Vim-specific T-cells compared to placebo. Additionally, within 28 days of DEN-181 administration, the percentage of CII-specific programmed cell death 1+ T-cells increased compared to placebo. Exploratory analysis revealed that improved RA disease activity was associated with the expansion of CII-specific and Cit-Vim-specific T-cells, a reduction in ACPA VDG, memory B cells, and inflammatory myeloid populations, and an enrichment in CCR7+ and naive T-cells. Furthermore, single-cell sequencing identified T-cell transcripts that were linked to tolerogenic TCR signaling and exhaustion after low or medium doses of DEN-181. Thus the safety and immunomodulatory activity of low/medium DEN-181 doses provide rationale to further assess antigen-specific immunomodulatory therapy in ACPA+RA.

Immune-Stromal Cell Crosstalk

Communication between immune and stromal cells plays a central role in the development of RA and psoriatic arthritis (PsA). However, the specific nature of these interactions within the synovial pathology of these two pathotypes can vary. The task of identifying the communication between immune and stromal cells at the site of inflammation in RA and PsA presents a challenge [24]. This study represents the first comprehensive transcriptomic analysis of the inflamed joint in RA and PsA, aiming to investigate the interactions between immune and stromal cells in the pathogenesis of synovial inflammation.

This investigation encompassed the examination of individual cellular transcriptomes of 178000 cells found within synovial tissue. The cells were obtained from five patients diagnosed with PsA and four patients diagnosed with RA. Importantly, the cells were not previously sorted into immune and stromal cell categories. This methodology allowed for a comprehensive transcriptomic analysis of the synovial tissue as a whole, leading to the identification of interactions between immune and stromal cells. Cutting-edge techniques in data integration and annotation were employed to identify and characterize 18 stromal cell clusters and 14 immune cell clusters.

A comprehensive examination of the gene expression profiles of different subsets of synovial cells reveals that the synovial T-cells are actively proliferating. Furthermore, the usage of λ and κ immunoglobulin light chains suggests that the synovial plasma cells may not originate from the local memory B-cell population. It is worth noting that the transcriptomic analysis of fibroblast and endothelial cell populations reveals distinct patterns, indicating the presence of various subgroups in patients with RA and PsA that are characterized by different usage of transcription factors. By investigating receptor-ligand interactions and downstream targets, we have identified a specific synergy between transforming growth factor (TGF)-β derived from synovial T-cells and interleukin (IL)-1β produced by macrophages in driving the transcriptional profile of invasive synovial fibroblasts expressing FAPα+THY1+. These fibroblasts are expanded in RA compared to PsA. In vitro experiments with synovial fibroblasts from patients with RA have demonstrated a metabolic shift toward glycolysis, increased expression of intercellular adhesion molecule 1, and secretion of IL-6 in response to combined treatment with TGF-β and IL-1β. Targeting specific interactions between immune and stromal cells provides promising avenues for therapeutic intervention in RA and PsA.

Challenges and opportunities

Despite the potential of innovative therapeutic methodologies, there are numerous obstacles that hinder the translation of these therapies from preclinical research to clinical practice. One of the primary hindrances is the variability in symptoms and disease progression observed among patients with RA. Due to the highly heterogeneous nature of RA, it becomes arduous to devise a universally applicable treatment strategy. Certain patients may exhibit positive responses to specific medications, while others may not. This variation poses significant challenges in devising the most suitable treatment plan for each individual.

An additional obstacle pertains to the potential adverse effects and risks associated with RA medications. Although DMARDs and biologics have demonstrated efficacy in symptom management, they concurrently elevate the likelihood of infections, liver toxicity, and other undesirable events. Achieving a balance between the advantages and disadvantages of these medications necessitates meticulous monitoring and customized treatment plans.

Access to healthcare and cost pose significant challenges in the treatment of RA. The accessibility of biologic medications, in particular, may be limited due to their exorbitant expense, thereby impeding their availability for certain patients. Moreover, the availability of specialized rheumatology care may be restricted in specific geographical regions, resulting in delayed diagnosis and treatment.

Moreover, progressions in technology and telemedicine present possibilities for distant surveillance and administration of RA. Portable gadgets and smartphone applications provide the capability for immediate monitoring of symptoms, physical activity, and adherence to medication. Telemedicine systems facilitate distant consultations and subsequent evaluations, augmenting the availability of specialized healthcare services for individuals with restricted mobility or residing in remote regions.

## Conclusions

The management of RA encounters difficulties in terms of the variability of symptoms, adverse effects of medications, and limited availability of healthcare. The future of innovative therapeutic methods in the treatment of RA holds great potential for patients who do not respond sufficiently to current therapies. Small molecule inhibitors, targeted biologics, cell-based therapies, and gene editing technologies present captivating prospects for enhancing the outcomes of the disease. Furthermore, progress in medical research presents opportunities for the identification of biomarkers and the implementation of personalized medicine in the treatment of RA. The capacity to discern specific indicators that signify the severity of the disease or the response to treatment can facilitate the customization of treatment plans for individual patients. This approach has the potential to yield more efficacious and streamlined treatment strategies, thereby minimizing the trial-and-error process of identifying the appropriate medications for each patient.

Nonetheless, rigorous clinical trials and further research are imperative to establish the safety, effectiveness, and cost-effectiveness of these pioneering approaches. By surmounting these obstacles and capitalizing on the opportunities, novel therapeutic strategies possess the capability to revolutionize the management of RA and offer improved outcomes for patients.

## References

[1] Guo Q, Wang Y, Xu D, Nossent J, Pavlos NJ, Xu J (2018). Rheumatoid arthritis: pathological mechanisms and modern pharmacologic therapies. Bone Res.

[2] Xu Y, Wu Q (2021). Prevalence trend and disparities in rheumatoid arthritis among US adults, 2005-2018. J Clin Med.

[3] Ahmed S, Jacob B, Carsons SE, De Leon J, Reiss AB (2021). Treatment of cardiovascular disease in rheumatoid arthritis: a complex challenge with increased atherosclerotic risk. Pharmaceuticals (Basel).

[4] Bello AE, Perkins EL, Jay R, Efthimiou P (2017). Recommendations for optimizing methotrexate treatment for patients with rheumatoid arthritis. Open Access Rheumatol.

[5] Curtis JR, Singh JA (2011). Use of biologics in rheumatoid arthritis: current and emerging paradigms of care. Clin Ther.

[6] Schwartz DM, Kanno Y, Villarino A, Ward M, Gadina M, O'Shea JJ (2017). JAK inhibition as a therapeutic strategy for immune and inflammatory diseases. Nat Rev Drug Discov.

[7] Harden RN, McCabe CS, Goebel A, Massey M, Suvar T, Grieve S, Bruehl S (2022). Complex regional pain syndrome: practical diagnostic and treatment guidelines, 5th edition. Pain Med.

[8] El-Saadony MT, Yang T, Korma SA (2022). Impacts of turmeric and its principal bioactive curcumin on human health: pharmaceutical, medicinal, and food applications: a comprehensive review. Front Nutr.

[9] Marengo MF, Suarez-Almazor ME (2015). Improving treatment adherence in patients with rheumatoid arthritis: what are the options?. Int J Clin Rheumatol.

[10] Wu H, Chen Y, Zhu H, Zhao M, Lu Q (2019). The pathogenic role of dysregulated epigenetic modifications in autoimmune diseases. Front Immunol.

[11] Perła-Kaján J, Jakubowski H (2019). Dysregulation of epigenetic mechanisms of gene expression in the pathologies of hyperhomocysteinemia. Int J Mol Sci.

[12] Ding Q, Hu W, Wang R (2023). Signaling pathways in rheumatoid arthritis: implications for targeted therapy. Signal Transduct Target Ther.

[13] Zhao H, Wu L, Yan G, Chen Y, Zhou M, Wu Y, Li Y (2021). Inflammation and tumor progression: signaling pathways and targeted intervention. Signal Transduct Target Ther.

[14] Bhandari S, Bhandari S, Bhandari S (2023). Chimeric antigen receptor T cell therapy for the treatment of systemic rheumatic diseases: a comprehensive review of recent literature. Ann Med Surg (Lond).

[15] Ghobadinezhad F, Ebrahimi N, Mozaffari F (2022). The emerging role of regulatory cell-based therapy in autoimmune disease. Front Immunol.

[16] Lee MH, Shin JI, Yang JW (2022). Genome editing using CRISPR-Cas9 and autoimmune diseases: a comprehensive review. Int J Mol Sci.

[17] Hasebe R, Murakami K, Harada M (2022). ATP spreads inflammation to other limbs through crosstalk between sensory neurons and interneurons. J Exp Med.

[18] Sakaguchi N, Takahashi T, Hata H (2003). Altered thymic T-cell selection due to a mutation of the ZAP-70 gene causes autoimmune arthritis in mice. Nature.

[19] McBride DA, Kerr MD, Johnson WT (2023). Immunomodulatory microparticles epigenetically modulate T cells and systemically ameliorate autoimmune arthritis. Adv Sci (Weinh).

[20] Suga K, Suto A, Tanaka S (2023). TAp63, a methotrexate target in CD4+T cells, suppresses Foxp3 expression and exacerbates autoimmune arthritis. JCI Insight.

[21] Bae S, Kim K, Kang K (2023). RANKL-responsive epigenetic mechanism reprograms macrophages into bone-resorbing osteoclasts. Cell Mol Immunol.

[22] Volkov M, van Schie KA, van der Woude D (2020). Autoantibodies and B cells: the ABC of rheumatoid arthritis pathophysiology. Immunol Rev.

[23] Sonigra A, Nel HJ, Wehr P (2022). Randomized phase I trial of antigen-specific tolerizing immunotherapy with peptide/calcitriol liposomes in ACPA+ rheumatoid arthritis. JCI Insight.

[24] Floudas A, Smith CM, Tynan O (2022). Distinct stromal and immune cell interactions shape the pathogenesis of rheumatoid and psoriatic arthritis. Ann Rheum Dis.

